# Structural Basis for the Ubiquitin-Linkage Specificity and deISGylating Activity of SARS-CoV Papain-Like Protease

**DOI:** 10.1371/journal.ppat.1004113

**Published:** 2014-05-22

**Authors:** Kiira Ratia, Andrew Kilianski, Yahira M. Baez-Santos, Susan C. Baker, Andrew Mesecar

**Affiliations:** 1 Department of Medicinal Chemistry and Pharmacognosy, University of Illinois, Chicago, Illinois, United States of America; 2 Department of Microbiology and Immunology, Loyola University Chicago Stritch School of Medicine, Maywood, Illinois, United States of America; 3 Department of Biological Sciences, Purdue University, West Lafayette, Indiana, United States of America; Institut Pasteur, France

## Abstract

Severe acute respiratory syndrome coronavirus (SARS-CoV) encodes a papain-like protease (PLpro) with both deubiquitinating (DUB) and deISGylating activities that are proposed to counteract the post-translational modification of signaling molecules that activate the innate immune response. Here we examine the structural basis for PLpro's ubiquitin chain and interferon stimulated gene 15 (ISG15) specificity. We present the X-ray crystal structure of PLpro in complex with ubiquitin-aldehyde and model the interaction of PLpro with other ubiquitin-chain and ISG15 substrates. We show that PLpro greatly prefers K48- to K63-linked ubiquitin chains, and ISG15-based substrates to those that are mono-ubiquitinated. We propose that PLpro's higher affinity for K48-linked ubiquitin chains and ISG15 stems from a bivalent mechanism of binding, where two ubiquitin-like domains prefer to bind in the palm domain of PLpro with the most distal ubiquitin domain interacting with a “ridge” region of the thumb domain. Mutagenesis of residues within this ridge region revealed that these mutants retain viral protease activity and the ability to catalyze hydrolysis of mono-ubiquitin. However, a select number of these mutants have a significantly reduced ability to hydrolyze the substrate ISG15-AMC, or be inhibited by K48-linked diubuiquitin. For these latter residues, we found that PLpro antagonism of the nuclear factor kappa-light-chain-enhancer of activated B-cells (NFκB) signaling pathway is abrogated. This identification of key and unique sites in PLpro required for recognition and processing of diubiquitin and ISG15 versus mono-ubiquitin and protease activity provides new insight into ubiquitin-chain and ISG15 recognition and highlights a role for PLpro DUB and deISGylase activity in antagonism of the innate immune response.

## Author Summary

All coronaviruses such as the SARS virus and the recently identified Middle East Respiratory Syndrome (MERS) virus encode in their genomes at least one papain-like protease (PLpro) enzyme that has two distinct functions in viral pathogenesis. The first function is to process the viral polyprotein into individual proteins that are essential for viral replication. The second function is to remove ubiquitin and ISG15 proteins from host cell proteins, which likely helps coronaviruses short circuit the host's innate immune response. The 3-dimensional structure of SARS virus PLpro in complex with a human ubiquitin analog was determined and reveals how coronavirus PLpro enzymes strip ubiquitin and ISG15 from host cell proteins at the molecular level. A series of amino acid residues involved in interactions between PLpro and ubiquitin were mutated to identify which interactions are important only for the recognition of ubiquitin and ISG15 modified proteins by PLpro and not for recognition and cleaving of the viral polyprotein. The 3D structure of SARS PLpro with ubiquitin-aldehyde sheds significant new light into how PLpro interacts with ubiquitin-like molecules and provides a molecular road map for performing similar studies on other deadly coronaviruses such as MERS.

## Introduction

Ubiquitin (Ub), a 76-amino-acid protein, is the building block for a set of versatile, post-translational modifications that regulate a number of cellular pathways, including many processes associated with combating viral infection [1]. Through the action of activating and conjugating enzymes, the C-terminus of ubiquitin is covalently attached to the ε-amino group of lysine side chains on target proteins, forming an isopeptide bond. The most common ubiquitin modifications are extended to form chains of ubiquitin molecules, linked through a lysine side chain on the proximal ubiquitin and the C-terminus of the neighboring distal ubiquitin. The complexity associated with ubiquitin modifications arises from the ubiquitin lysine residue participating in the polyubiquitin chain. There are 7 lysine residues on ubiquitin, and most are believed capable of forming homotypic chains that mediate different linkage-dependent cellular pathways [2]. The most prevalent ubiquitin linkage and most widely studied, the K48-based chain, directs the modified protein to the proteasome for degradation [3]. Another well characterized Ub modification, the K63-based chain, is commonly associated with regulating endocytic processes, the DNA-damage response, and innate immune response pathways [2]. More recently, the recognition of linear ubiquitin chains has been implicated in the activation of NFκB signaling [4].

In addition to the many possible modifications associated with ubiquitin, a structurally homologous family of proteins comprised of the ubiquitin superfold, the ubiquitin-like proteins (UBLs), can also be conjugated to target proteins to mediate different responses [5]. Examples of ubiquitin-like tags include the SUMO family of proteins, NEDD8, FAT10, and ISG15. Of interest to this study is the interferon-stimulated gene 15 (ISG15), which is comprised of two tandem ubiquitin-like folds. Conjugation of ISG15 to target proteins (ISGylation) is dramatically up-regulated following cellular stimulation by interferons or viral infection [6].

As effector molecules, ubiquitin chains and UBLs such as ISG15 must be recognized by downstream components, through protein-protein interactions, to elicit the appropriate cellular response. The differentiation of various linkages and UBLs by these interacting proteins is particularly important since the nature of the ubiquitin and UBL linkages dictates the downstream response. Recognition of a specific chain type can be accomplished by various ubiquitin binding domains [7], with one or several of these domains coexisting on a ubiquitin/UBL-interacting protein. As with all other post-translational modifications, ubiquitin chains and UBLs can also be removed from modified proteins by deconjugating enzymes [8], thereby reversing the effect of the modification on the protein. The human genome encodes approximately 100 deubiquitinating (or deubiquitylating) enzymes (DUBs) and other deconjugating enzymes (e.g. deISGylating) with specificities ranging from linkage-specific to target-specific to promiscuous [8]. The structural basis for how a DUB or downstream Ub/UBL-binding partner deciphers such subtly different modifications is of particular interest. In recent years, a number of cellular and viral proteins have been identified that exploit or interfere with cellular ubiquitin/UBL modifications [9], [10], [11], [12], [13]. Structural analysis of cellular and viral DUBs in association with ubiquitin chains has revealed the molecular basis for interaction with specific Ub-linkages [14], [15], [16]. Distinct sites on both host (USP21) and viral DUBs (vOTU) were shown to mediate interaction between the DUB and various Ub-linkage chains. Recently, exploiting knowledge of the interacting residues to guide mutagenesis studies allowed researchers to engineer the equine arterivirus ovarian tumor domain (vOTU) DUB enzyme with tailor-made specificity to either polyubiquitin or ISG15 [17]. Here, we explore this strategy to uncouple the viral protease polyprotein processing activity of the papain-like protease (PLpro) of Severe Acute Respiratory Syndrome Coronavirus (SARS-CoV) from its DUB and deISGylating activities.

SARS-CoV emerged into the human population in 2002–2003 from a bat reservoir to cause a pandemic infecting more than 8000 people with a case/fatality ratio of 10% [18]. The Middle East Respiratory Syndrome cornonavirus (MERS-CoV) also recently entered the human population and has infected over 130 individuals and so far has killed over 58 individuals. Identifying targets for antiviral therapies and viral factors that contribute to delayed or reduced innate immunes responses are important for developing strategies to control CoV infections. PLpro, required for proteolytic processing of the SARS-CoV replicase polyprotein [19], [20], structurally resembles the ubiquitin specific protease (USP) class of human deubiquitinating enzymes (DUBs) [21], [22] and has been shown to have both deubiquitinating and deISGylating activities [23], [24], [25]. Though the exact cellular targets for these accessory activities are unknown, PLpro has been directly linked to suppressing interferon-β (IFNβ) production by the innate immune response during virus infection [26]. More specifically, pathways leading to the activation of both IRF3 and NFκB, essential transcription factors of the *ifnb* gene, are blocked in the presence of PLpro [27], [28]. Both K48- and K63-linked ubiquitin chains, as well as ISG15 modifications, have been shown to play important roles in regulating IRF3 and NFκB activation [29]. Here we investigate the molecular recognition of monoubiquitin, K48- and K63-linked ubiquitin chains, and ISG15 by SARS-CoV PLpro, and test whether disrupting these interactions alters antagonism of the innate immune response.

## Results

### The structure of PLpro bound to ubiquitin aldehyde

**Figure 1:**
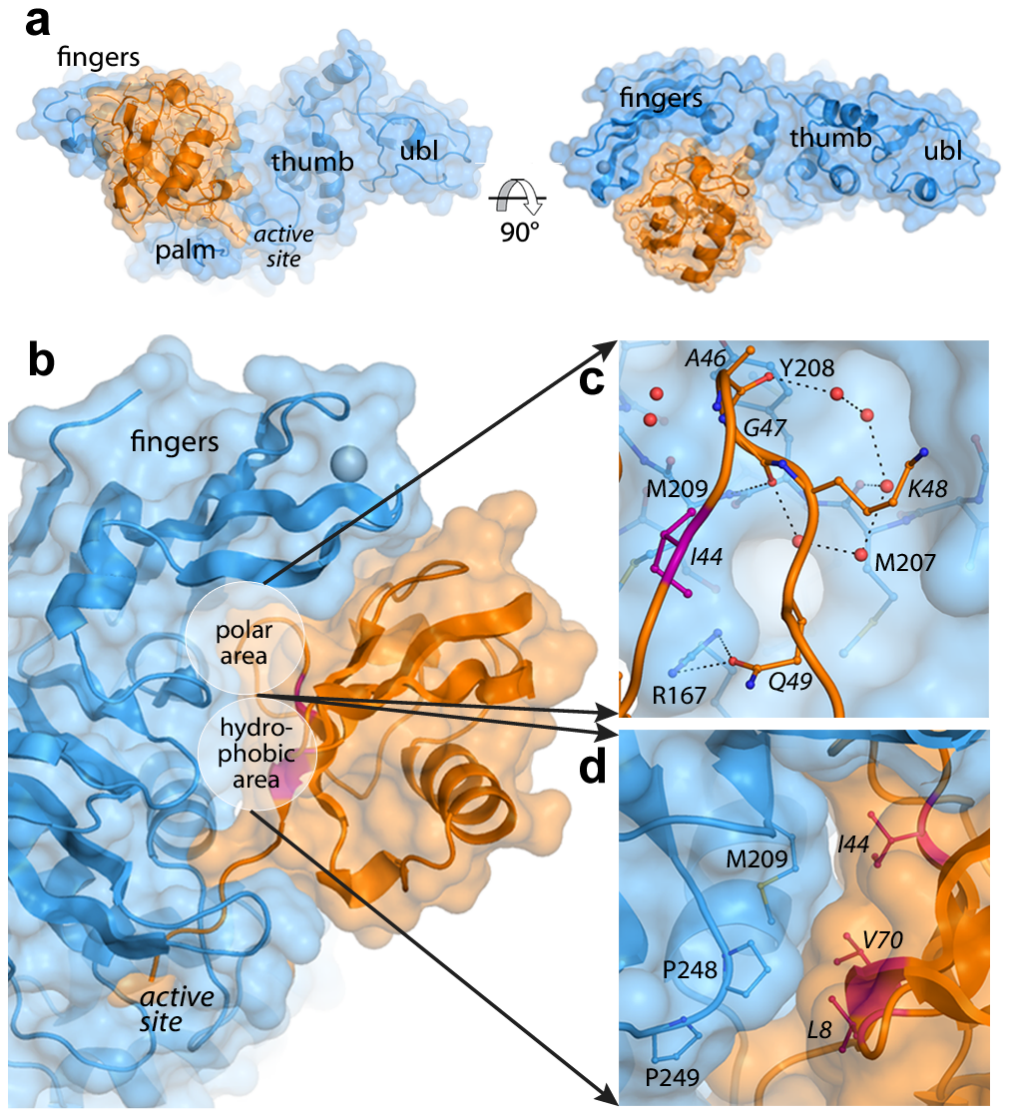
The PLpro-ubiquitin interface involves both polar and hydrophobic interactions. PLpro is shown in blue and ubiquitin is shown in orange. Areas of interest are labeled. The ‘hydrophobic patch’ of ubiquitin is colored in magenta. Ubiquitin residues are italicized. **A.** Surface representation of PLpro bound to ubiquitin aldehyde. **B**. Overall schematic of the interface. **C.** A detailed view of the polar area of contacts designated in (B). Hydrogen bonds directly between PLpro and ubiquitin and those between intervening water molecules (red spheres) are shown as dashed lines. **D**. A detailed view of the hydrophobic area of contacts designated in (B).

Due to the structural and functional similarities of PLpro with human deubiquitinating enzymes [21], we sought to examine the structural requirements for ubiquitin recognition by PLpro via X-ray crystallography. In order to co-crystallize PLpro with ubiquitin, a semisynthetic version of ubiquitin containing a C-terminal aldehyde functional group was employed for crystallization. Ubiquitin aldehydes (Ubals) can modify the catalytic cysteine of deubiquitinating enzymes in a covalent but reversible manner, and can account for a 300,000-fold increase in binding affinity relative to unmodified ubiquitin [30]. The PLpro-Ubal structure was determined to a resolution of 2.75 Å (Table S1), with one PLpro and one Ubal monomer comprising the asymmetric unit. The substrate-free (unbound) structure of PLpro, which we previously described, presents a large, unobstructed binding surface for ubiquitin, but access to the enzyme's active site is blocked by a flexible, glycine-hinged β-turn [21]. As expected for ubiquitin-specific proteases, the body of ubiquitin makes contacts with the palm and fingers regions of PLpro (Figure 1A), with little perturbation to the overall enzyme structure. Due to the considerably smaller size of PLpro's palm and fingers regions relative to those of its human USP counterparts, HAUSP (USP7) and USP14, the ubiquitin molecule buries much less surface area upon binding to PLpro, forming a 900 Å^2^ protein-protein interface, compared to a 3600 Å^2^ HAUSP-ubiquitin interface [31].

The interactions at the PLpro-Ubal protein-protein interface are comprised of van der Waals contacts and both direct and water-mediated hydrogen bonds (Figure 1B). As observed for HAUSP [31] and USP2 [32] interactions with ubiquitin, a number of water molecules line the protein-protein interface including a loop of four ubiquitin residues (A46-Q49) that is involved in a number of polar interactions with the palm of PLpro (Figure 1C). Directly adjacent to this loop, the hydrophobic patch of ubiquitin (I44, V70, and L8), often the focal point of many ubiquitin-protein interactions [33], makes extensive contacts with the side chains of M209 and P248 on PLpro (Figure 1D).

**Figure 2:**
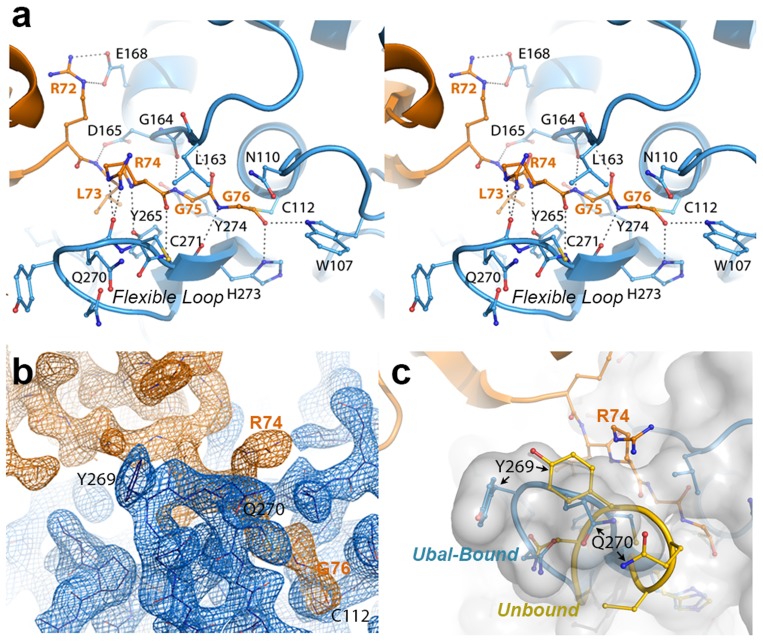
The crystal structure of the PLpro-Ubal complex reveals a dense hydrogen-bonding pattern between the active site of PLpro and the C-terminus of ubiquitin. **A.** Stereoview of PLpro active-site interactions with ubiquitin-aldehyde. PLpro residues are shown in blue and labeled in black, and ubiquitin residues are shown and labeled in orange. Hydrogen bonds between PLpro and ubiquitin are shown as dashed lines. **B.** Electron density associated with the region surrounding the C-terminal residues of ubiquitin (orange density) and their interactions with the PLpro active site in the region of the mobile loop (blue density). The residues shown and the view depicted are similar to those in panels A and C. The electron density maps were calculated by omitting ubiquitin from the structure factor calculations. The Fo-Fc map for ubiquitin (orange) is contoured at 3σ and the 2Fo-Fc map for PLpro (blue density) is contoured at 1.5σ. The figure was generated using the program Pymol. **C.** Comparison of the PLpro active site loop in bound (blue with white surface) and unbound (yellow) conformations. The C-terminus of ubiquitin is shown in orange. The orientation of the structure is similar to that shown in panels A and B.

Despite the complete burial of PLpro's palm domain and a significant portion of the body of ubiquitin, the vast majority of PLpro-ubiquitin interactions involve the 5 C-terminal residues of ubiquitin (R72-G76). In this area, 12 intermolecular hydrogen bonds align and direct the ubiquitin C-terminus to the catalytic cysteine (C112) in the active site of PLpro (Figure 2A). The flexible beta-turn loop that blocks the tunnel to the PLpro active site in the unbound enzyme undergoes a significant conformational change and becomes significantly more ordered as it interacts with the C-terminus of ubiquitin. The electron density associated with this loop is well resolved (Figure 2B) and the motion of the loop opens the active site of PLpro to accommodate and to hydrogen bond to the side chain of R74 on ubiquitin (Figure 2C). The large number of hydrogen bonds and other contacts involved in the interaction between PLpro and the 5 C-terminal residues of ubiquitin (RLRGG) suggest that a significant amount of binding energy is contributed by these 5 amino acids of ubiquitin. Indeed, the 5-amino acid peptide RLRGG-AMC is hydrolyzed by PLpro with a *k*_cat_/K_m_ = 5.5×10^3^ M^−1^ s^−1^. However, the K_m_ value for this peptide substrate is too large to measure experimentally, and the catalytic rate of hydrolysis of this substrate is approximately 13.6-fold lower than that of ubiquitin-AMC (*k*_cat_/K_m_ = 7.5×10^4^ M^−1^ s^−1^) [25]. Therefore, other regions of PLpro distant from the active site are important for the binding and catalysis of ubiquitin and UBL substrates.

### PLpro prefers K48- to K63-linked ubiquitin and ISG15 to mono-ubiquitin

**Figure 3:**
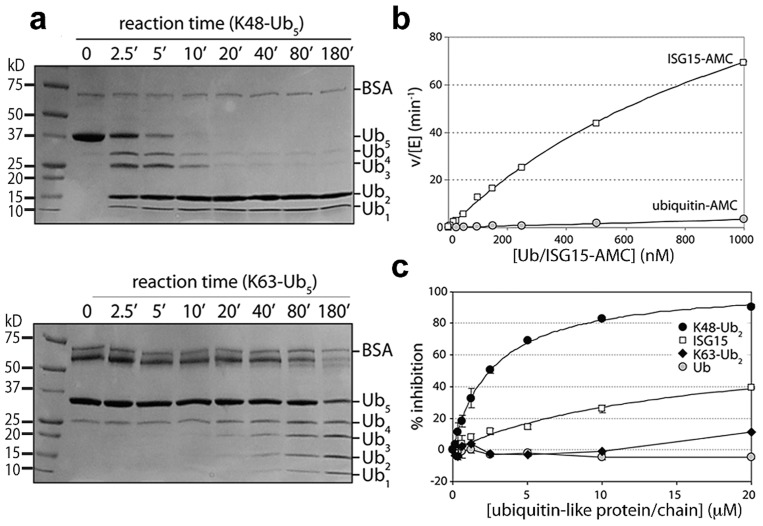
PLpro prefers K48-linked ubiquitin to K63-linked ubiquitin and ISG15 to mono-ubiquitin. **A.** SDS-PAGE analysis of PLpro isopeptidase activity with K48-Ub_5_ (top gel) and K63-Ub_5_ (bottom gel) chains. Reactions times (in minutes) are indicated above each well, and the different ubiquitin species are marked to the right of the gels. Note the inability of PLpro to process K48-Ub_2_ to mono-ubiquitin. **B.** Comparison of PLpro activity with Ub-AMC (gray circles) and ISG15-AMC (white squares) as a function of substrate concentration. **C.** The inhibition of PLpro activity by free mono-ubiquitin (gray circles), free ISG15 (white squares), K63-Ub_2_ (black diamonds) and K48-Ub_2_ (black circles) is plotted as a function of inhibitor concentration.

Though PLpro recognizes and cleaves ubiquitin well, presumably at a faster rate than it cleaves its own viral polyprotein substrate [25], further analysis of PLpro catalyzed reaction kinetics reveals that it has a 30- to 50-fold greater specificity for another ubiquitin-like modifier, ISG15 [24] (Figure 3), a molecule consisting of two tandem ubiquitin-like domains involved in modulating the innate immune response. Although PLpro has a measurable rate of hydrolysis activity towards Ub-AMC over a concentration range of 1000 nM, it cannot be saturated indicating that mono-ubiquitin interacts weakly with the enzyme (Figure 3B). In contrast, the ISG15-AMC substrate is hydrolyzed at a much more significant rate than Ub-AMC and saturation of SARS-CoV PLpro is observable suggesting that ISG15-AMC is the preferred substrate in terms of catalytic efficiency.

To further probe the recognition and catalysis of different ubiquitin and UBL proteins by PLpro, we performed a series of kinetic studies to determine the ability of PLpro to hydrolyze/deconjugate a series of different polyubiquitin chains (Figure 3A) and to hydrolyze the AMC group from the substrates Ub-AMC and ISG15-AMC (Figure 3B). In addition, we determined whether some of the products of these reactions (Ub, ISG15, K48-Ub2 and K63-Ub2) would serve as inhibitors of PLpro (Figure 3C). The results of a time-course comparison between PLpro cleavage of K48- and K63-linked pentaubiquitin chains (Ub_5_) demonstrate that PLpro rapidly degrades K48-linked chains, with only moderate activity towards K63-linked chains (Figure 3A). The processing of linear tetra-ubiquitin chains by PLpro was also investigated but no activity was observed under any of the experimental conditions tested (Figure S1). PLpro is therefore able to distinguish between K48-linked, K63-linked and linear polyubiquitin chains, with a significant preference for K48 linkages.

Despite PLpro's strong preference for processing K48-Ub chains, the rapid breakdown of K48-Ub_5_ into its smaller Ub_x_ components does not follow an unbiased trend. Initially, PLpro rapidly cleaves K48-Ub_5_ into Ub_4_, Ub_3_, Ub_2_ and Ub species. Rather surprisingly though, the Ub_4_ and Ub_3_ products continue to be rapidly converted to mono- and di-ubiqutin but the levels of Ub_2_ do not diminish significantly over 3 hours (Figure 3A, top gel). Di-ubiqutin remains a primary product of the K48-Ub_5_ cleavage reaction even following extended incubation suggesting that the generation of Ub_2_ during chain cleavage leads to product inhibition. These initial observations led to the hypothesis that PLpro preferentially recognizes a diubiquitin species on the distal side of the isopeptide bond of K48-linked polyubiquitin chains, a pattern that does not extend to K63-based chains (Figure 3A, bottom gel). To further explore this hypothesis, we next determined the extent of inhibition of PLpro hydrolysis of the RLRGG-AMC substrate by mono-Ub, K48-Ub_2_, K63-Ub_2_ and ISG15 (Figure 3C). Over a concentration range of 20 µM, PLpro does not bind to K63-Ub_2_ or to mono-ubiquitin with sufficient affinity to inhibit PLpro activity. However, ISG15 displays moderate inhibition, and K48-Ub_2_ dramatically inhibits PLpro activity (IC_50_ = 2.7±0.2 µM), implying that ISG15 and K48-Ub_2_ bind to PLpro significantly more tightly than mono-Ub and K63-Ub_2_.

### Inhibition of PLpro by K48-Ub_2_ and ISG15, but not K63-Ub_2_, supports a bidentate recognition mechanism

**Figure 4:**
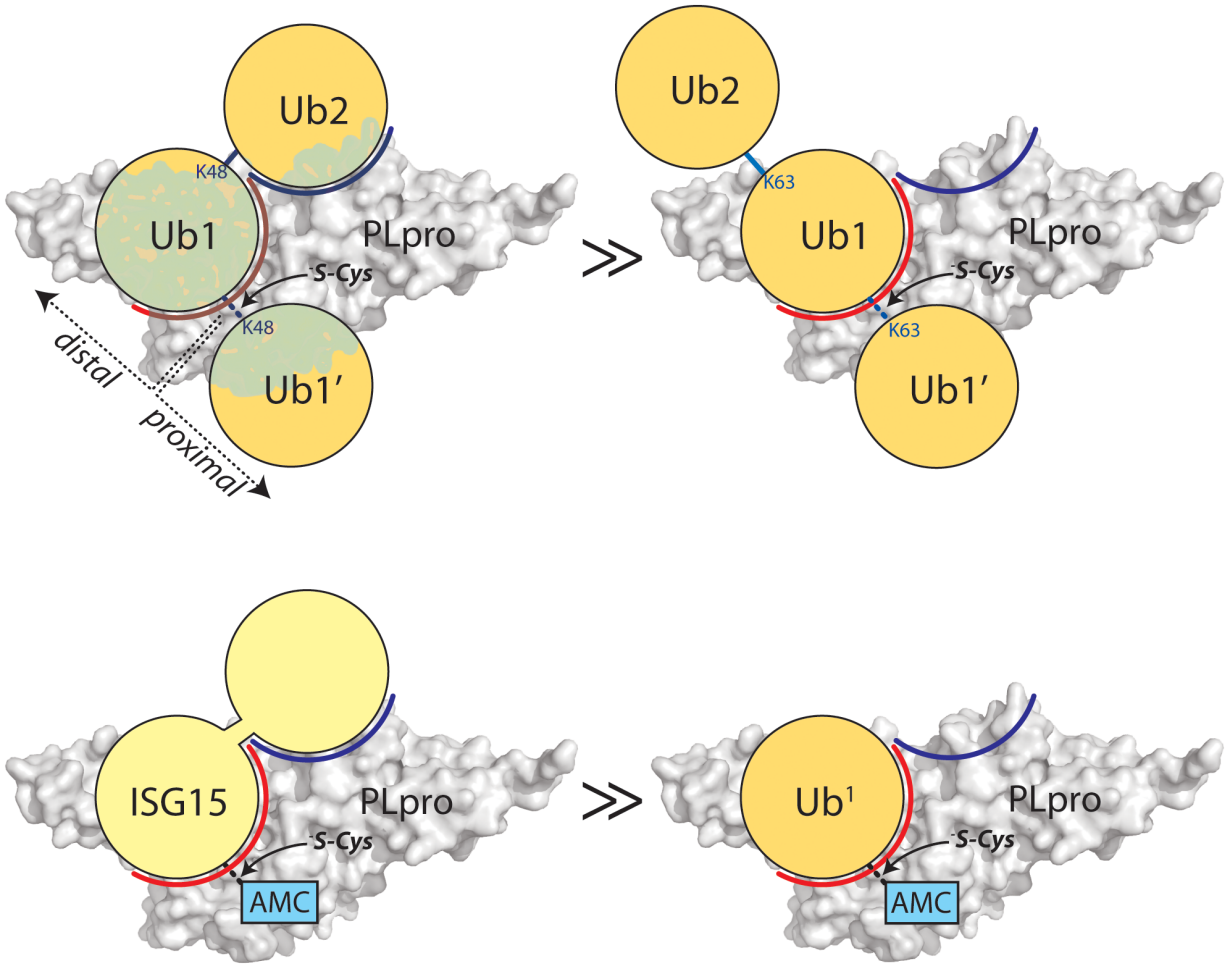
Proposed recognition models of K48-Ub_2_ and ISG15 by PLpro. PLpro is shown as a surface representation and colored grey. The ubiquitin binding subsites distal to the isopeptide bond are indicated in red for SUb1 and blue for SUb2. Ubiquitin molecules in the chain are indicated as circles in yellow and numbering follows conventional protease substrates numbering with ubiquitins distal to the isopeptide bond as Ub2, Ub1 and those proximal as Ub1′ etc. Ubiquitin lysines are labeled as K48 or K63. The greater than symbols (>>) designate the relative affinity of one complex over the other from data presented in Figure 3.

With the structure of PLpro bound to ubiquitin aldehyde in hand, we sought to elucidate the potential binding modes of K48-Ub_2_ and ISG15 to PLpro. Because ISG15 closely resembles a di-ubiquitin molecule (Figure S2), we hypothesized that PLpro may have a second or extended binding site that recognizes a second ubiquitin or ubiquitin-like domain of ISG15 or K48-Ub_2._ These potential binding or recognition sites for polyubiquitin chains on SARS-CoV PLpro are illustrated schematically in Figure 4. We have chosen here to use nomenclature similar to that used for describing how proteases recognize their peptide substrates. Each amino acid of the substrate distal to the peptide cleavage site is designated P1, P2, P3 etc, and each amino acid proximal to the cleavage site is designated P1′, P2′, P3′ etc. The recognition subsites for each of these amino acids on the protease are designated S1, S2, S3 etc and S1′, S2′ S3′ etc. Here, we define each ubiquitin or ubiquitin-like domain distal to the isopeptide cleavage site as Ub1, Ub2, Ub3 etc, and each ubiquitin or ubiquitin-like domain proximal to the isopeptide cleavage site as Ub1′, Ub2′ etc. We also define each recognition site or surface on PLpro as either SUb1, SUb2 etc.

**Figure 5:**
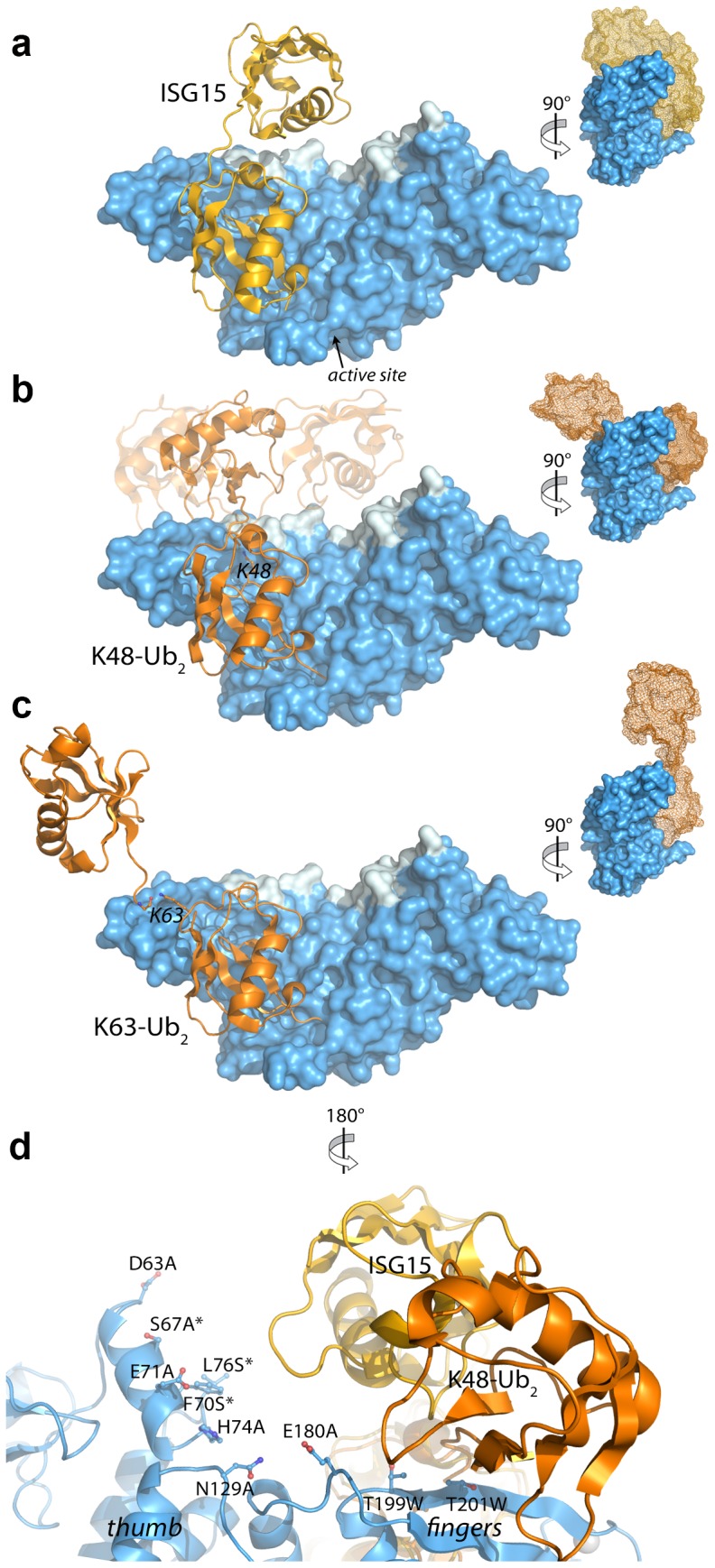
Modeling and mutational analysis suggest that PLpro binds to ISG15 and K48-Ub_2_, but not K63-Ub_2_, in a bidentate manner. Initial model of PLpro (blue surface) bound to ISG15 (yellow) (**A**), K48-Ub_2_ (orange) (**B**), and K63-Ub_2_ (orange) (**C**). The multiple conformations of the distal ubiquitin depicted as transparent cartoons in (B) represent the range of pre-experimental binding configurations for K48-Ub_2_. **D.** A detailed view of mutated PLpro residues designed to locate the second binding site for K48-Ub_2_ and ISG15. These residues are shaded in cyan in **A–D**. Residues marked with an asterisk designate follow-up mutations.

We established in the studies presented in Figure 3 that PLpro greatly prefers the binding of K48-Ub_2_ over mono-ubiquitin and that this recognition must stem from a portion of the second ubiquitin molecule (Ub2). Previous work by Lindner *et al.* [24] showed that removal of the N-terminal ubiquitin-like domain (equivalent to Ub2) of ISG15-AMC leads to a 6-fold loss in SARS-CoV PLpro recognition of ISG15, verifying that this N-terminal domain contributes major determinants to the specificity of PLpro for ISG15. This information, in conjunction with the knowledge that PLpro does not bind with appreciable affinity to K63-Ub_2_ (Figure 3C), provided the initial parameters for constructing molecular models of PLpro in complex with ISG15 and K48-Ub_2_ (Figure 5).

Because the crystal structures of K48-linked [34], [35] and K63-linked [36], [37] ubiquitin chains and of ISG15 [38] have been reported, a simple molecular alignment of these structures with the Ubal molecule bound to PLpro reveals important similarities between K48-Ub_2_ and ISG15 relative to K63-Ub_2_ (Figures 5A, 5B and 5C). It is widely established that K63-linked ubiquitin chains form an extended, almost linear, conformation [36], [37], whereas K48-linked chains have a proclivity for more compact arrangements [34], [35]. The positioning of K63 on the PLpro-bound Ubal directs the second ubiquitin molecule (Ub2) of a diubiquitin chain away from the body of PLpro (Figure 5C), particularly if the K63 chain remains in the favored extended conformation. This K63-linked model prevents further interactions between the second ubiquitin (Ub2) and its SUb2 subsite (region indicated by the blue curved line in Figure 4) on PLpro. Conversely, the position of K48 on the PLpro-Ubal structure, buried in the palm domain of PLpro, dictates that any diubiquitin chain will likely make extensive contacts with PLpro at both the SUb1 and the SUb2 subsites, though the isopeptide bond joining the two ubiquitin molecules (Ub1 and Ub2) is too flexible to restrict the positioning of the second Ub2 molecule to a predefined SUb2 of PLpro (Figure 5B). Finally, the structure of ISG15 is more rigid than K48-Ub_2_, and direct alignment of the C-terminal domain of ISG15 (equivalent to Ub1 of a diubiquitin chain) with PLpro-Ubal projects the N-terminal domain of ISG15 (equivalent to the Ub2 of a diubiquitin chain) onto a “ridge” defining the perimeter of the fingers domain of PLpro (Figure 5B), which is the potential SUb2 subsite. This ridge region or SUb2 subsite, the white shaded residues in Figures 5A, 5B and 5C, can be accessed by the Ub2 domains of both K48-Ub_2_ and ISG15.

### K48-Ub_2_ and ISG15 interact with a region on the thumb domain of PLpro

**Figure 6:**
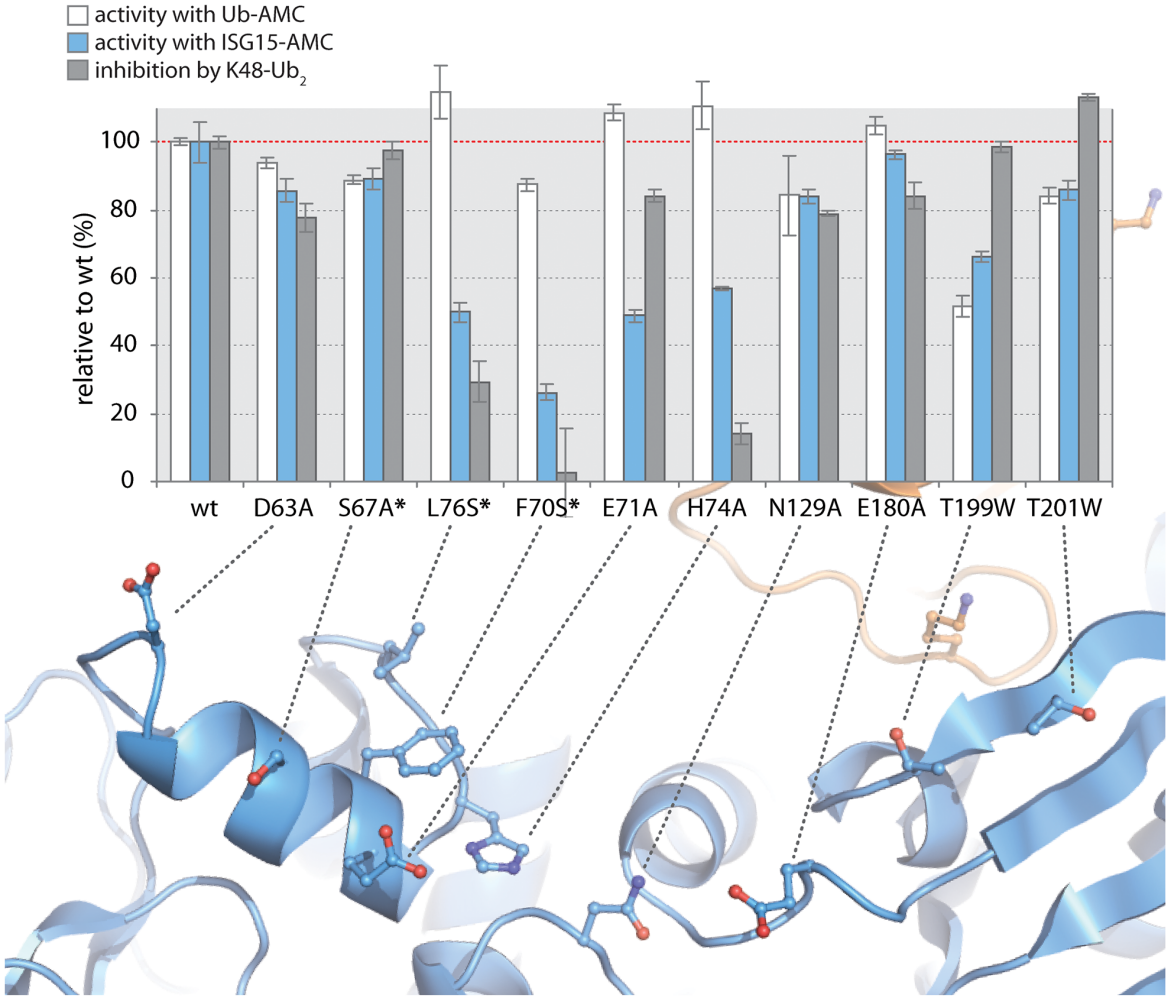
K48-Ub_2_ and ISG15 interact with a “ridge” region on the thumb domain of PLpro. PLpro mutants were purified and tested for activity with Ub-AMC (white bars) and ISG15-AMC (blue bars) and for their ability to be inhibited by 5 µM K48-Ub_2_ (gray bars). All measurements were performed in triplicate and are reported in % activity or % inhibition relative to wild-type PLpro. The ribbon diagram below the bar graph illustrates the position of each mutant on PLpro. Mutants marked with an asterisk represent follow-up mutants.

To test the validity of these initial models proposed in Figure 5, and to further refine the positioning of ISG15 and K48-Ub_2_ on the structure of PLpro, we surveyed the uppermost region of the SUb2 ridge of PLpro for amino acids that potentially interact with ISG15 or K48-Ub_2_. Seven amino acids were chosen “to cast a wide net” over this potential SUb2 binding surface with the purpose of perturbing ISG15 or K48-Ub_2_ interactions with PLpro through regional scanning, site-directed mutagenesis (Figure 5D). To ensure that the mutations did not alter the catalytic activity of PLpro or its ability to bind mono-ubiquitin, all purified mutants were first tested for their catalytic activity towards RLRGG-AMC and Ub-AMC substrates (Figure 6). Mutants that retained high activity towards these substrates, but lost catalytic activity with ISG15-AMC and/or lost the ability to be inhibited by K48-Ub_2_, were further investigated (Figure 6). Most notably, two mutants, E71A and H74A, were fully active with Ub-AMC but their ability to either hydrolyze ISG15-AMC or be inhibited by K48-Ub_2_ was perturbed (Figure 6). The E71A mutation caused a 50% drop in activity with ISG15-AMC but did not affect the inhibitory potency of K48-Ub_2_, whereas the H74A mutant had 58% activity with ISG15-AMC, relative to wild-type PLpro, and was significantly less inhibited by K48-Ub_2_ (14% inhibition) than wild-type (100% inhibition).

The positioning of residues E71 and H74 on PLpro (Figure 5D) suggests that the N-terminal half of ISG15 (equivalent to Ub2) and Ub2 of K48-Ub_2_ are interacting with a helical region (residues D63 to H74) at the top of the thumb domain of PLpro. To test this hypothesis, three additional residues in this region were mutated to more accurately define the area of contact between PLpro and the two ligands (Figure 6, starred mutants). Two of these mutants, F70S and L76S, greatly affected both ISG15 and K48-Ub_2_ binding without causing detrimental effects to catalysis of Ub-AMC (Figure 6, starred mutants). F70 and L76 together comprise a solvent-exposed, hydrophobic patch on the thumb domain of PLpro that is neighbored by E71 and H74, also shown to contribute to binding of ISG15 and K48-Ub_2_ (Figure 6). The retention of Ub-AMC hydrolysis activity and loss of recognition for the second ubiquitin or ubiquitin-like domain (Ub2) is a significant result as the decoupling of these two sites has yet to be achieved in other USP enzyme systems and signifies that it is achievable. The biological significance of decoupling diubiquitin from monoubiquitin recognition was explored next.

**Figure 7:**
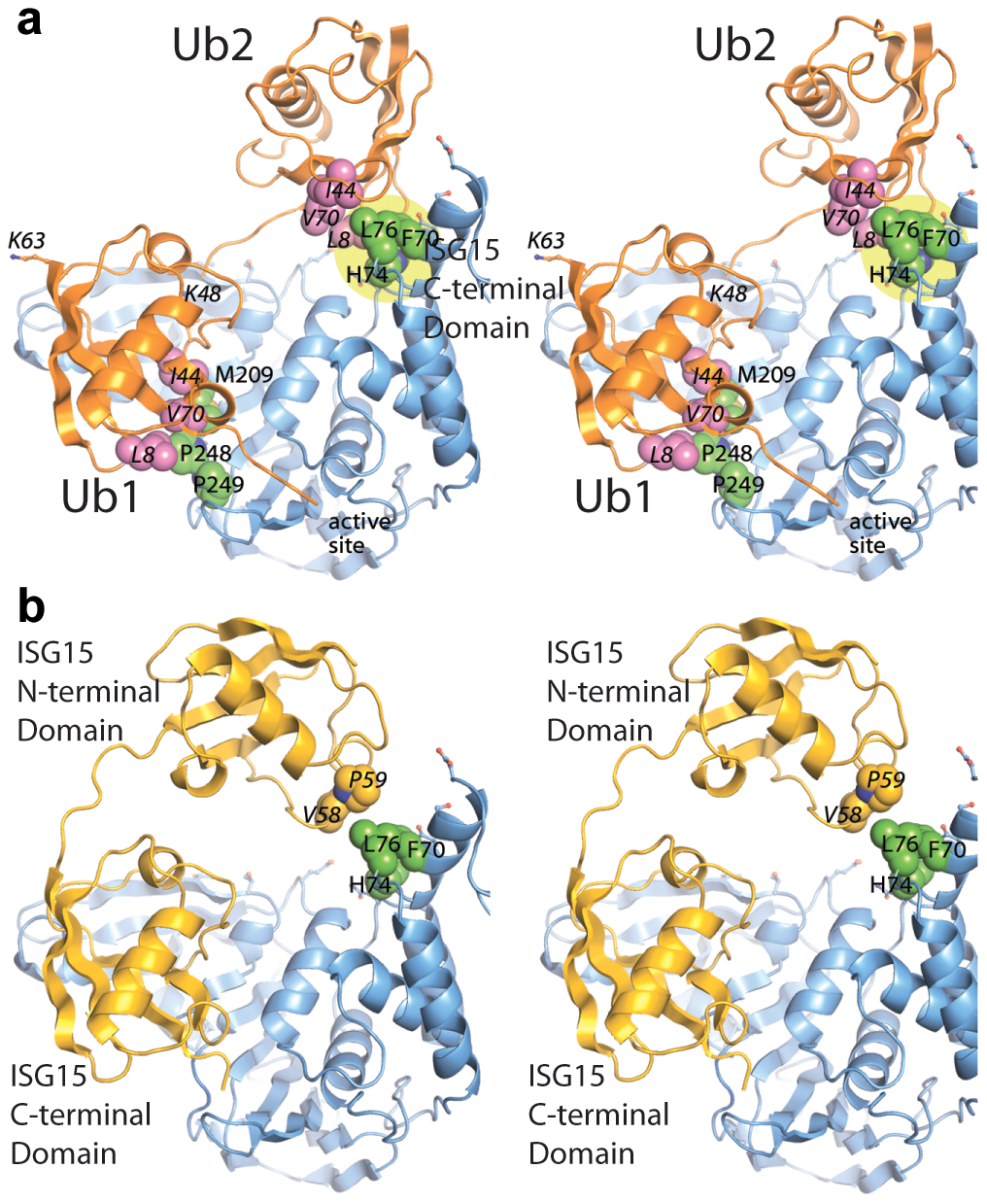
Updated models of (A) ISG15 (yellow) and (B) K48-Ub_2_ (orange) and bound to PLpro (blue). The ubiquitin hydrophobic patch residues are shown in magenta. PLpro residues identified through site-directed mutagenesis as important for K48-Ub_2_ and ISG15 binding are highlighted in a yellow circle. The two distal regions involved in binding Ub2 or ISG15 are labeled as distal-1 (closest to active site, location of single ubiquitin binding) and distal-2 (binding of second ubiquitin-like domain).

The mutant analysis allowed further refinement of our structural models of PLpro bound to ISG15 or K48-Ub_2_, providing an established area of contact on PLpro. Both models were manually edited and refined to reflect a closer association on the thumb domain of PLpro (Figure 7). The N-terminus of ISG15 was shifted towards the thumb and rotated slightly about the flexible linker connecting the N- and C-terminal domains to present a small hydrophobic region on ISG15 (V58-P59) to the complementary surface (F70, L76) on the PLpro thumb (Figure 7B). Because there is little information on the conformational flexibility of ISG15, only conservative manipulations of the structure were made. Conversely, K48-Ub_2_ has been shown to adopt numerous conformations [39]. Because the hydrophobic patch of Ub (I44, V70, and L80) is ostensibly critical to the majority of interactions between ubiquitin and its binding partners [40], an effort was made to align this patch in the more distal ubiquitin of K48-Ub_2_ with the identified hydrophobic patch on the PLpro thumb domain (Figure 7A). The structural model is able to position and accommodate this interaction well, further supporting the results of the mutagenesis studies.

### PLpro ridge mutants in the SUb2 subsite are impaired in their ability to deubiquitinate proteins and antagonize the NFκB pathway in the host cell

**Figure 8:**
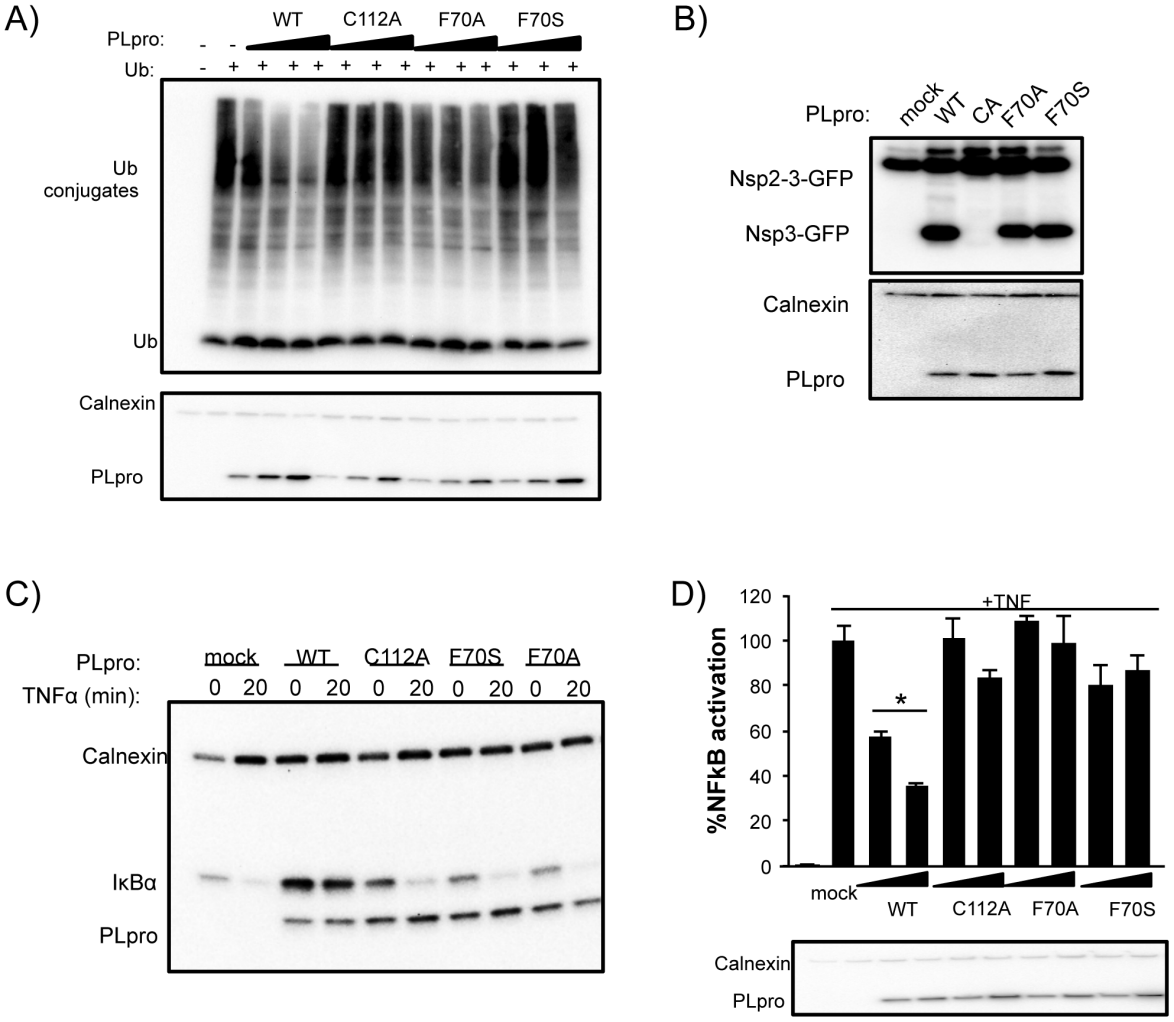
Deubiquitinating activity and NF-κB antagonism are reduced by mutation of SARS-CoV PLpro residue F70. (**A**) HEK293T cells were transfected with expression plasmids encoding FLAG-Ub and the indicated PLpro. At 18 hours post transfection, cells were lysed and immunoblotted for FLAG-Ub, calnexin, and PLpro-V5. (B) HEK293T cells were transfected with constructs expressing nsp2-3-GFP and SARS-CoV PLpro-V5. Lysates were immunoblotted with anti-GFP, anti-calnexin, and anti-V5. (C) HEK293 cells were transfected with IkBα-HA and the indicated PLpro. After 16 hours incubation, cells were stimulated with TNFα (20 ng/ml). Lysates were analyzed by 10% SDS-PAGE and immunoblotted for anti-HA, anti-calnexin, and anti-V5. (D) 293HEK cells were transfected with NFkB-reporter and Renilla luciferase control constructs and the indicated PLpro. After 12 hours, TNFα was added to a final concentration of 10 ng/mL and the cells were incubated for an additional 4 hours. Results are normalized to induction of NFkB reporter activity by TNFα. Panels below are western blots of the lysates using anti-V5 for detection of PLpro and anti-actin as a protein loading control. Experiments were performed in triplicate and repeated twice. * = p<.05 statistical difference from mock transfected cells by student t-test.

To determine if PLpro mutants, particularly at residue F70, affect PLpro deubiquitinating activity, we analyzed this activity in transfected cells (Figure 8A). Wild-type PLpro cleaves ubiquitin from host cells in a dose dependent manner. In contrast, mutation of the catalytic cysteine (C112A) or residue F70 (F70A and F70S) within the ridge region of the SUb2 subsite show a reduction in the dose-dependent deconjugation of ubiquitin from host cell substrates. These data agree with the loss in affinity of PLpro for Ub_2_ and ISG15 by this and other ridge mutants *in vitro* (Figure 6). The ability of the F70S and F70A PLpro mutants to recognize and cleave the viral polyprotein was determined next by testing for their ability to cleave a substrate in a trans-cleavage assay [27]. Importantly, both the F70S and F70A ridge mutants retained protease activity at similar levels to WT PLpro, as demonstrated by the ability to process the nsp2-3 polyprotein substrate (Figure 8B and Figure S3A). As expected, the control C112A mutant was unable to process the nsp2-3 polyprotein.

Previous studies documented PLpro as an antagonist of the NFκB signaling pathway [27], [41]. We sought to determine if mutations in PLpro that alter interactions with K48-Ub affect the K48-polyUb-mediated degradation of IκBα induced upon activation of the NFκB pathway by TNFα [42]. 293T cells were transfected with plasmid DNA encoding IκBα-HA and either PLpro WT or the F70S and F70A ridge mutants. At 16 hours post transfection, cells were treated with TNFα to induce ubiquitination and degradation of IκBα, and protein levels of IκBα and PLpro were monitored after 20 minutes by western blotting (Figure 8C). As expected, IκBα is rapidly degraded in mock-infected cells. In contrast, IκBα is more abundant in cells expressing PLpro and there is no detectable degradation of IκBα after treatment with TNFα. Interestingly, the response of cells to TNFα treatment in the presence of either the C112A catalytic mutant or the F70S or F70A ridge mutants of PLpro were essentially the same as mock-infected cells. Similar data and results were obtained with each individual ridge mutant or with combinations of ridge mutants (Figure S3). These results are consistent with an inability of these mutants to either hydrolyze the isopeptide bonds (C112A) or bind ubiquitin chains (F70S or F70A) such as K48-Ub_2_ and ISG15 or *in vitro* (Figure 5) and support a model whereby the deubiquitinating activity of WT PLpro is responsible for removing K48-linked Ub from IκBα thereby preventing signaling of NFκB.

To further investigate the effect of mutation of the ridge domain on antagonism of the NFκB mediated activation of transcription, we evaluated PLpro WT, the C112A catalytic mutant, and both the F70A and F70S mutant PLpro enzymes in a dual-luciferase reporter assay containing an NFκB response element regulating transcription of firefly luciferase (Figure 8D). In mock-transfected cells, the NFκB-dependent reporter is potently activated by the addition of media containing TNFα. This activation is antagonized in a dose-dependent manner when cells are transfected with different concentrations of wild-type PLpro (Figure 8D). In contrast, the PLpro C112A catalytic mutant and F70S mutants are unable to effectively antagonize the NFκB pathway. These results, combined with the IκBα degradation assay described above, are consistent with a mechanism by which SARS-CoV PLpro blocks NFκB activation, and suggest that the ridge-region of the PLpro SUb2 subsite is important for the antagonism of the NFκB pathway.

## Discussion

Distinguishing the roles of multifunctional enzymes in viral replication is challenging. A critical first step is the proof-of-principle that an enzyme has distinct binding sites for different substrates. For SARS-CoV PLpro, an enzyme with peptidase and isopeptidase activities, the X-ray structure of PLpro in complex with ubiquitin aldehyde, and subsequent modeling of ubiquitin-chain and ISG15 interactions, suggested that a ridge region in PLpro is likely involved in binding ubiquitin-like modifier substrates. Analysis of PLpro mutants in protease and isopeptidase (DUB and DeISGylase) activity and competitive binding assays identified a hydrophobic portion of this ridge region in the SUb2 subsite that is essential for robust deISGylaing activity and interactions with ubiquitin chains, but is not required for protease or Ub-AMC hydrolysis activity. Thus, we were able to successfully decouple the viral protease processing activity of PLpro from its ubiquitin-chain and ISG15 hydrolysis activities.

The use of co-crystal structures to identify ubiquitin and ISG15 interacting regions and subsequent re-engineering of enzymatic activity has also been applied to the study of cellular DUBs such as USP21, and to another class of viral proteases that also have DUB activity such as the ovarian tumor (OTU) domains of Crimean Congo Hemorrhagic Fever Virus (CCHFV) [14], [15], [16]. Analysis of USP21 with linear diubiquitin revealed an ubiquitin-specific SUb2 (S2) binding site that, when re-engineered, weakened interactions with diubiquitin and reduced the ability of the enzyme to remove ISG15 from cellular substrates [14]. Co-crystal structure studies of the viral OTU domain from CCHFV revealed a critical hydrostatic interaction with ISG15. Mutagenesis of vOTU residues interacting with ISG15, specifically Q16R [15] or E128A [16], resulted in enzymes that were dramatically reduced in the ability to hydrolyze ISG15 while retaining the ability to hydrolyze polyubiquitin. Thus, co-crystal structure analysis of multifunctional enzymes can reveal novel sites that allow for separation of enzymatic activity and allow for targeted development of therapeutics.

The structure of PLpro bound to ubiquitin-aldehyde illustrates how the multi-domain architecture of an enzyme can distinguish different ubiquitin chains and ubiquitin-like proteins by forming the support structure for two separate ubiquitin binding domains. For PLpro, these binding sites are in the palm domain (SUb1) for the interaction with Ub1 (red shaded region in Figure 4) and a hydrophobic patch, termed the “ridge” domain (SUb2) for the interaction with Ub2 (blue shaded region in Figure 3D). The canonical right-hand domain layout of ubiquitin-specific proteases (USPs) allows for a large, primary ubiquitin binding surface on the palm and finger domains, with the C-terminus of ubiquitin firmly coordinated in the active site through numerous intermolecular hydrogen bonds [8]. In this conformation, the hydrophobic patch of Ub1 is well positioned to interact with hydrophobic residues on the palm and fingers of PLpro (Figure 1). This general mode of binding is relatively conserved across known structures of USPs with [8], though the tilt of Ub relative to its C-terminus, or size of the protein-protein interface, can be influenced by accessory loops, or by the relative sizes of the domains themselves. These factors also dictate the environment of different ubiquitin lysine residues, and thus the potential for a USP to bind a particular type of ubiquitin linkage internally. USP14, a human deubiquitinating enzyme associated with the proteasome [43], is specific for K48-linked Ub chains, which it removes from proteins immediately prior to their degradation in the proteasome. USP14 is comprised of a much larger fingers domain relative to that of PLpro, which may obstruct extended K63 Ub chains from binding and being cleaved by USP14 [44]. Conversely, CYLD, a human DUB that negatively regulates the NF-kB pathway, is specific for K63-based Ub chains. CYLD contains a truncated fingers domain, which is expected to accommodate extra ubiquitin moieties extending from K63 of the primary ubiquitin [45].

It has been shown that certain proteins capable of distinguishing different ubiquitin chains do so at the region around the linkage [4], [46]. In the case of the DUBs, this recognition would occur at the scissile isopeptide bond in the active site, or rather how the proximal ubiquitin (Ub1′) is oriented at the exit of the active site (Figure 4). Assuming equivalent binding of Ub1 at the SUb1 subsite, UB1′ would be presented differently to the SUb1′ subsite of a DUB depending on the linkage. AMSH-LP, a zinc-dependent DUB that regulates receptor trafficking, favors K63-Ub linkages by recognizing the particular ubiquitin surface around K63 of Ub1′, allowing for correct alignment of the isopeptide bond in the active site [46]. Though it is not directly clear if SARS-CoV PLpro favors cleavage of one type of linkage over the other at the active site, our results suggest that the binding of ubiquitin species in at least two Ub subsites (SUb1 and SUb2) located distal to the isopeptide bond in the active site are crucial for recognition. Due to the inhibitory nature of K48-Ub_2_ (Figure 3C), we can infer that this species binds more tightly to the SUb2-SUb1 subsites instead of the SUb1-SUb1′ subsites since the concentration levels of K48-Ub_2_ increase during the hydrolysis of K48-linked ubiquitin chains causing product inhibition and hence the reduction of cleavage of K48-Ub_2_ to mono-ubiquitin. Otherwise, the K48-Ub_2_ species would be expected to be efficiently cleaved to monoubiquitin (Figure 3A). Thus, K48-Ub_2_ strongly favors occupation of the SUb2-SUb1 subsites of SARS-CoV PLpro (red and blue shaded regions in Figure 4) in favor of spanning the Ub1-Ub1′ binding sites.

The activity of SARS-CoV PLpro towards Ub-AMC rivals those of the most active human DUBs that have been characterized so far. For example, UCH-L3 [47] has a *k*_cat, Ub-AMC_ value of 9.1±0.1 s^−1^, and USP8 [48] has a *k*_cat, Ub-AMC_ value of 2.4 s^−1^ [14], [15]. SARS-CoV PLpro is highly active towards the substrate ISG15-AMC (Figure 3B) with a *k*_cat,ISG15-AMC_ value of 6.2±0.3 s^−1^. This turnover rate is so far unmatched by any other human or viral DUB studied to date. The most active vOTU is from the Crimean-Congo hemorrhagic fever virus with a *k*_cat, ISG15-AMC_ = 0.54±0.05 s^−1^ [49], [50] and the most active USP so far is human USP21 with a *k*_cat, ISG15-AMC_ = 5.9×10^−3^±1.3×10^−3^ s^−1^. We attempted to determine the kinetic rates of hydrolysis of ISG15-AMC by the human deISGylase USP18(UBP43), but the rate was at least 10^3^ slower than that with PLpro, which is consistent with other studies. Though the exact outcome of PLpro's deISGylating activity during viral infection is currently unknown, ISG15 is an important activator of the immune response. Recent studies have shown that ISG15 is conjugated to newly synthesized proteins, a potential cellular strategy to disrupt the function of viral proteins, which are abundantly produced during infection [51]. SARS-CoV and other viruses, including nairo- and arteriviruses [52], may have evolved deISGylating strategies to counteract such defenses. ISG15 has also been shown to positively regulate IRF3 activation by preventing an inhibitor, Pin1, from binding to IRF3 [53]. SARS-CoV PLpro, which has been shown to prevent IRF3 activation [26], may deISGylate IRF3 or bind to IRF3 through a conjugated ISG15 molecule to prevent its activation.

It is intriguing to speculate that the DUB activity of SARS-CoV PLpro is important either for viral replication or pathogenesis. SARS-CoV PLpro is extremely efficient at deconjugating K48-linked chains and has measurable activity with K63-based chains. K48-based Ub conjugation has been demonstrated to be an important positive activator of the NFκB pathway [54]. Moreover, K63-based chains have been shown to be key components of the innate immune response as they mediate protein complex formation [1]. Three human DUBs, CYLD, A20 and DUBA, have been shown to negatively regulate the innate immune response by removing K63-based chains [55], [56], and USP15 inhibits the NFκB pathway by removing K48-Ub from IκBα, preventing its degradation [57]. We examined the effect of mutations within the ridge of PLpro on NFκB antagonism (Figures 8 and S3). IκBα is phosphorylated and degraded in response to TNFα, an activator of the NFκB pathway. The presence of PLpro prevents this degradation, and leads to an increase in basal levels of IκBα. These data indicate that PLpro is preventing the degradation and normal turnover of IκBα. The F70 ridge mutants (F70A and F70S) of SARS-CoV PLpro are unable to prevent the degradation of IκBα, suggesting that the K48-linked deubiquitinating activity of PLpro is required for PLpro's ability to antagonize the NFκB pathway. Importantly, Frieman et al. have shown that PLpro does not block TNFα stimulated IκBα phosphorylation [27] supporting a model whereby the stabilization of cellular IκBα is achieved through its K48-linked deubiquitination by PLpro. By separating the protease and isopeptidase activities of SARS-CoV PLpro, we have shown that K48-linked ubiquitin interaction is a mechanism behind the NFkB antagonism functions of SARS-CoV PLpro and that viral deubiquitinating enzymes might be critical determinants of pathogenesis.

The recent outbreak of the new MERS-CoV [58], a coronavirus that produces SARS-like symptoms in patients and has a high mortality, emphasizes the importance of continuing studies on the pathogenesis of deadly coronaviruses. MERS-CoV encodes a single papain-like protease (PLpro) similar to SARS-CoV, but the primary structure is more homologous to the bat coronavirus HKU5 (Figure S4). Comparing the amino acid sequences of SARS-CoV and MERS-CoV, specifically the amino acids that were mutated in this study and shown to have an effect on ubiquitin-like modifier recognition (Figure 6), reveals that there is little to no sequence conservation among these residues in the ridge region. This observation suggests that MERS-CoV PLpro is likely to recognize and process ubiquitin and ISG15 substrates differently than SARS-CoV PLpro. Since these residues are in the ridge region of the SUb2 subsite and are involved in binding Ub2, the differences are most likely to be found in ubiquitin-like chain recognition. It will be interesting to compare the polyubiquitin chain recognition and cleavage properties of MERS-CoV to SARS-CoV PLpro to test this hypothesis.

To date no FDA-approved vaccines or antivirals are available for human coronaviral infections, so information about SARS-CoV virulence factors are of critical importance [59]. Attenuation of SARS-CoV by affecting PLpro DUB activity provides a novel strategy in SARS-CoV and SARS-like coronavirus vaccine development. Directly targeting PLpro using antivirals would not only prevent polyprotein cleavage, but could also inhibit the innate immune antagonist functions of PLpro. Furthermore, understanding the molecular basis for ubiquitin chain interactions with cellular DUBs is an emerging strategy for developing specific human USP inhibitors that may be useful in the treatment of cancer, neurologic and infectious diseases [60].

In summary, we have determined the structure of a viral protease, SARS-CoV PLpro, in complex with ubiquitin aldehyde and have used this structure in conjunction with kinetic studies on the interaction of PLpro with Ub chains and ISG15 to guide molecular modeling. The resulting models of PLpro bound to two high affinity ligands, K48-Ub_2_ and ISG15, suggest that contacts with these ligands extend beyond the mono-ubiquitin binding site (SUb1). Site-directed mutagenesis studies identified a region of the thumb domain of PLpro as a second ubiquitin binding site (SUb2) for binding both K48-Ub_2_ and ISG15, but not K63-Ub_2_, a weaker ligand. Finally, these mutations are relevant to PLpro's function as an innate immune antagonist, as PLpro ridge mutants lose the ability to block NFκB-mediated signaling. Re-engineering of these PLpro mutants or others in the context of SARS-CoV may reveal a role for PLpro DUB activity in viral pathogenesis.

## Methods

### Synthesis of ubiquitin aldehyde

Ubiquitin aldehyde (Ubal) was synthesized using the intein-fusion method as previously described [61] with minor modifications. Expression of ubiquitin (residues 1–75) fused to intein and chitin-binding domains (plasmid pTYB2-Ub_1–75_) was carried out in BL21(DE3) Codon+ cells (Novagen), using LB auto-induction media [62] supplemented with 50 µg/mL carbenicillin. Following 24 h of growth at 25°C, 3 L of culture were pelleted, resuspended in Buffer A (25 mM HEPES, pH 6.8, 50 mM sodium acetate, 75 mM NaCl) and lysed by addition of Triton X-100 to 0.16% followed by sonication. The lysate was clarified by centrifugation and loaded onto a gravity-flow column containing 50 mL of chitin beads (New England Biolabs) equilibrated with Buffer A. Following a series of column washes with Buffer A to remove unbound proteins, 25 mL of Buffer A containing freshly prepared 100 mM sodium 2-mercaptoethanesulfonate (MESNA) (Sigma-Aldrich) was added to the resin to create a slurry. The slurry was incubated overnight at 4°C with gentle rocking. The following day, the resin was separated from the effluent, which contained released ubiquitin thioester, and washed with 80 mL of Buffer A to fully elute any remaining released ubiquitin. Samples containing ubiquitin thioester were combined, concentrated to 20 mL (1–3 mg/mL), and dialyzed against 5 mM HCl overnight at 4°C.

Ubiquitin acetal was generated by reacting 10 mL of ubiquitin thioester with 2 mL of 4M aminoacetaldehyde diethyl acetal, pH 8.5 (Sigma-Aldrich) and 50 µL of freshly prepared 2M N-hydroxy-succinimide (Sigma-Aldrich) and incubated at room temperature. Upon full conversion (∼1 hr), the ubiquitin acetal was dialyzed against 50 mM sodium acetate, pH 4.5, overnight at 4°C. The acetal was deprotected with 0.15 M HCl to produce ubiquitin aldehyde, then quenched with 0.15 M Tris base, and subsequently desalted into 20 mM HEPES, pH 7.5. Conversions from thiol ester to acetal and from acetal to aldehyde were monitored by HPLC on a C8 column as previously described [61]. The concentrations of the ubiquitin species throughout the purification were determined by HPLC, using unmodified ubiquitin (Boston Biochem) as a standard.

### Purification and crystallization of PLpro-ubiquitin aldehyde complex

The catalytic domain of SARS-CoV PLpro, polyprotein residues 1541–1855, was expressed and purified as previously described [25]. To form the PLpro-Ubal complex, 30 mg of purified PLpro was added to 11 mg of Ubal in 20 mM HEPES, pH 7.5, and the complex was incubated overnight at 4°C. The activity of PLpro was monitored to ensure full inactivation of the protease by Ubal. The resulting complex was purified from unreacted Ubal and hydrolyzed ubiquitin, a byproduct of aldehyde synthesis, using a 10/100 GL Mono-Q column (GE Healthcare) at pH 7.5. Fractions containing PLpro-Ubal were pooled and concentrated to 10–15 mg/mL. PLpro-Ubal crystals were grown by vapor diffusion at room temperature from hanging drops containing 1 µL of protein complex (3–12 mg/mL PLpro-Ubal in 20 mM Tris, pH 7.5) and 3 µL of precipitant (0.1 M HEPES, pH 7.5, 10% isopropanol, 20% PEG 4,000). Crystals, which grew within two days, were flash-frozen in liquid nitrogen without the need for additional cryo-protectants, and then transferred to a SPINE puck system for X-ray data collection at the Advanced Photon Source of Argonne National Laboratory.

### X-ray data collection and structure refinement

X-ray data were collected at LS-CAT on beamline 21-ID-F to 2.76 Å and resulted in an overall R_merge_ value of 6.3% with 98% completeness. Data were processed and scaled using the HKL2000 program suite [63], and crystals of PLpro-Ubal were indexed to the P3_1_21 space group with unit cell dimensions of a = b = 47.12 Å and c = 332.56 Å, with one monomer in the asymmetric unit. Phases for the complex were obtained from a molecular replacement solution using a monomer of the unbound PLpro structure (PDB entry: 2FE8) and free ubiquitin (PDB entry: 1UBQ) as search models using Phaser [64], which identified one unique molecular replacement solution of the complex. Rigid body refinement followed by iterative rounds of restrained refinement and modeling building using the programs Refmac5 [65] and WinCoot [66] reduced the R-factors to 18.7% (R_cryst_) and 27.9% (R_free_). The final structure and associated structure factors have been deposited in the PDB under PDB ID 4MM3 and RCSB ID RCSB082081.

### Site-directed mutagenesis and purification of PLpro mutants

Site-directed mutations were introduced into a pET15b-PLpro construct using synthetic primers purchased from Integrated DNA Technologies and the QuikChange Site-Directed Mutagenesis Kit (Stratagene). All mutations were verified by sequencing. The pET15b-PLpro construct expresses an N-terminal His_6_-tag followed by the ubiquitin-like and catalytic domains of SARS-CoV PLpro (polyprotein residues 1541–1855). Wild-type and mutant versions of PLpro were expressed in BL21(DE3) cells and were purified using Ni-NTA agarose (Qiagen). Enzyme concentration was determined using the Bradford protein assay.

### Ubiquitin chain cleavage experiments

Proteolytic cleavage of homogeneous, K48-linked or K63-linked penta-ubiquitin (Boston Biochem) was carried out under the following conditions: 0.07 µg of purified PLpro (23 nM) was incubated with 50 µg of K48-Ub_5_ or K63-Ub_5_ at 25°C in an 85 µL volume containing 50 mM HEPES, pH 7.5, 0.1 mg/mL BSA, 100 mM NaCl, and 2 mM DTT. A control reaction was incubated under identical conditions with the exclusion of enzyme. At various time points, 10 µL were removed and quenched with the addition of SDS-PAGE sample loading dye to a 1× concentration (25 mM Tris, pH 6.8, 280 mM β-mercaptoethanol, 4% glycerol, 0.8% SDS, 0.02% bromophenol blue), and heat treated at 95°C for 5 minutes. The samples were analyzed by electrophoresis on a 4–12% SDS-PAGE and stained with Coomassie dye.

### Measurement of PLpro inhibition by ubiquitin, ISG15, K48-Ub_2_ and K63-Ub_2_

Wild-type PLpro inhibition by free Ub, ISG15, K48-Ub_2_, and K63-Ub_2_ was measured in a 96-well plate format in duplicate at 25°C. Assays contained 50 mM HEPES, pH 7.5, 0.1 mg/mL BSA, 5 mM DTT, an unsaturating concentration of the substrate RLRGG-AMC (37.5 µM), 45 nM purified PLpro, and varying concentrations of Ub, ISG15, K48-Ub_2_, or K63-Ub_2_ (Boston Biochem). Reaction progress curves were monitored continuously using a Tecan Genios Pro plate reader, where free AMC fluorescence was measured at λ_excitation_ = 360 nm, λ_emission_ = 460 nm. Where applicable, the percent inhibition of the reaction was calculated relative to PLpro activity in the absence of the ligand. An IC_50_ value was calculated using the equation *v* = *a*/(1+([*I*]/*IC_5_*_0_) where *v* is the velocity of the reaction in the presence of inhibitor, [*I*] is the concentration of the inhibitor, *a* is the velocity of the reaction in the absence of inhibitor, and *IC_50_* is the concentration of inhibitor resulting in 50% inhibition of enzyme. Data were fit to the equation using the Enzyme Kinetics module of Sigmaplot (Systat Software).

### Measurement of substrate interactions with PLpro mutants

All reactions were carried out at 25°C in 50 mM HEPES, pH 7.5, 0.1 mg/mL BSA, and 5 mM DTT. AMC fluorescence was detected as above. Wild-type and mutant activities with 400 nM Ub-AMC and 400 nM ISG15-AMC were measured at 15 nM and 0.15 nM enzyme, respectively. Inhibition by K48-Ub_2_ was measured by comparing enzyme activity (at 63 nM) with 37.5 µM RLRGG-AMC in the absence and presence of 5 µM K48-Ub_2_.

### Protein-protein docking

The models of K48-Ub_2_, K63-Ub_2_, and ISG15 bound to PLpro were initially generated by superimposing the most proximal portions of each molecule onto the ubiquitin molecule in the PLpro-Ubal structure. The PDB files utilized for each model are as follows: ISG15 (1Z2M), K63-Ub_2_ (3H7S), K48-Ub_2_ (2KDF). Only the model of PLpro-K48-Ub_2_ required further editing, with the distal ubiquitin being manually manipulated about the isopeptide bond in Pymol to alleviate clashes with PLpro.

### In cellular deubiquitination assays

HEK293T cells in 12-well Cell-Bind plate (Corning) were transfected (LT1, Mirus) with 300 ng FLAG-Ub and either 125 ng, 250 ng, or 500 ng of pcDNA3.1-SARS-PLpro per well. Cells were incubated for 18 hours then lysed with IkBα lysis buffer (20 mM Tris (pH 7.5), 150 mM NaCl, 1 mM EGTA, 1 mM EDTA, 1% Triton X-100, 2.5 mM Na pyro-phosphate, 1 mM Beta-glycerophosphate, 1 mM Na ortho-vanadate, 1 ug/ml Leupeptin) and incubated for 20 min on ice. Lysates were subjected to centrifugation at 4C and the cytoplasmic contents added to 2× sample buffer and separated by SDS-PAGE on a 4–20% gradient gel (BioRad). Gel was transferred to PVDF using semi-dry apparatus (BioRad) and immunoblotted with anti-FLAG (Sigma), anti-V5 (Invitrogen), and anti-calnexin (BD).

### Trans-cleavage assays

HEK293 cells were transfected with constructs expressing nsp2-3-GFP and SARS-CoV PLpro-V5 wild type, catalytic mutant (C112A) or ridge mutants (F70A, F70S). Cells were incubated for 24 hours and then lysed with IkBα lysis buffer (20 mM Tris (pH 7.5), 150 mM NaCl, 1 mM EGTA, 1 mM EDTA, 1% Triton X-100, 2.5 mM Na pyro-phosphate, 1 mM Beta-glycerophosphate, 1 mM Na ortho-vanadate, 1 ug/ml Leupeptin). Lysates were subjected to centrifugation at 4C and the cytoplasmic contents added to 2× sample buffer and separated by SDS-PAGE on a 10% PAGE gel (BioRad). Samples were transferred to PVDF immunoblotted with anti-GFP (Life Technologies) and anti-V5 (Invitrogen).

### IkBα-HA degradation assay

HEK293 cells were plated in 60 mm dishes and transfected with 3 ug pcDNA3.1 – PLpro and 250 ng pIkBα-HA per dish (Mirus LT-1) [42]. Cells were incubated for 24 hours, media, removed, and fresh media containing TNFα (Roche) at a final concentration of 20 ng/ml was added and incubated with cells. Cells were incubated with TNFα for the indicated time points. Cells were then lysed with an IkBα lysis buffer: 20 mM Tris (pH 7.5), 150 mM NaCl, 1 mM EGTA, 1 mM EDTA, 1% Triton X-100, 2.5 mM Na pyro-phosphate, 1 mM Beta-glycerophosphate, 1 mM Na ortho-vanadate, 1 ug/ml Leupeptin. Lysates were kept on ice and centrifuged at 14000× g for 10 min at 4C. After centrifugation, supernatant was added to equal volume 2× sample buffer, boiled for 5 min, and then immediately run on 10% SDS-PAGE gel. Gel was transferred to PVDF using a semi-dry blot apparatus (TransBlot Turbo, Bio-Rad) and blocked in 5% milk overnight. Antibodies were used at a concentration of 1∶5000 (mαHA, Covance; mαV5, Invitrogen). Western blots were quantified using a Typhoon Image Scanner and ImageQuant5. p<.05 indicates a significant difference from WT PLpro transfected cells and was determined using a student t test with Systat.

### Dual-luciferase assay

HEK293 cells were plated in 24-well plates (Cell-Bind from Corning) and transfected with 50, 100, or 150 ng of pcDNA3.1 – PLpro plasmid, 50 ng pNFkB-luc, and 25 ng pRL-TK (Renilla control) per well in triplicate (Mirus LT-1). Cells were incubated for 12 hours, media removed, and fresh media containing TNFα (Roche) at a final concentration of 10 ng/ml was added and incubated with cells for 4 hours. Cells were then lysed with passive lysis buffer and wells assayed for dual luciferase expression (Promega) by luminometer. p<.05 indicates a significant difference from mock transfected cells and was determined using a student t test with Systat.

## Supporting Information

Figure S1 Processing of linear Ub_4_ by SARS-CoV PLpro. SARS-CoV PLpro (15 µM) was incubated with 3 µg of linear-Ub_4_ for 1 and 2 hours (h) at room temperature. The reaction was quenched with SDS sample buffer containing 250 mM Tris, pH 6.8, 10% SDS, 50% glycerol, 0.02% bromophenol blue and 35 mM beta-mercaptoethanol. Protein bands corresponding to Ub and BSA are shown. The molecular weight marker is shown in kD. No cleavage of linear Ub_4_ by SARS-CoV PLpro is detected.
(TIF)

Figure S2 ISG15 resembles a di-ubiquitin molecule. Overlay of K48-Ub_2_ (orange) and ISG15 (yellow) rendered a Cα RMSD of 6.32 Å (147 to 147 atoms). The two distal regions involved in binding Ub_2_ or ISG15 are labeled as Ub distal-1 (closest to active site, location of single ubiquitin binding) or ISG15 C-terminal domain and Ub distal-2 (binding of second ubiquitin-like domain) or ISG15 N-terminal domain.
(TIF)

Figure S3 (A) HEK293 cells were transfected with constructs expressing nsp2-3-GFP and SARS-CoV PLpro-V5 wild type, catalytic mutant (C112A) or ridge mutants. Cells were incubated for 24 hours at 37°C and then lysed with lysis buffer A. Lysates were run on 10% SDS-PAGE and Western blot was performed using anti-GFP and anti-V5. (B) Quantification of IκBα using Fluorchem E System and AlphaView software (Protein Simple). ** = The levels of IkB-HA were significantly increased in the presence of WT PLpro compared to mock, C112A, and F70S (p<0.05) by mixed ANOVA and there was no decrease after TNFα treatment (p = 0.675) by Dunnet t-test. (C) 293HEK cells were transfected with a construct containing a firefly luciferase reporter driven by an NFkB dependent promoter and a Renilla luciferase under control of a constitutive promoter. After 12 hours, TNFα was added to a final concentration of 10 ng/mL and the cells were incubated for an additional 4 hours. Cells were lysed in passive lysis buffer and 25 ul of lysate was used in Promega's Dual Luciferase Reporter Assay. Results are normalized to induction of NFkB reporter activity by TNFα. Panels below are western blots of the lysates using anti-V5 for detection of PLpro and anti-actin as a protein loading control. Experiments were performed in triplicate and repeated twice. * = p<0.05 statistical difference from mock transfected cells by student t-test.
(PDF)

Figure S4 Multiple sequence alignments presenting the secondary structure elements on top: α-helices (squiggles), β-strands (black arrows) and turn (TT). Highlighted are the highly conserved areas (blue boxes) containing the conserved residues (red boxes), homologous residues (red font), and divergent residues (black font). (A) Comparison of the amino acid sequence between the β-grasp domain of ubiquitin to each β-grasp domain of ISG15. The residues comprising the ubiquitin and ISG15 hydrophobic patch are highlighted with a magenta arrow and a yellow box, respectively. The structure elements were generated using the X-ray crystal structure of ubiquitin (pdb: 1UBQ). (B) The papain-like protease (PLpro) domain from the beta coronavirus 2b (SARS and bat-SARS), 2c (bat-HKU4 and 5) and 2d (HUK9) share high amino acid sequence homology. SARS PLpro residues identified by site-directed mutagenesis as important for K48-Ub_2_ and ISG15 binding are highlighted with green arrows, while those that did not seemed to be important are highlighted with blue arrows. The α-helix 2 (highlighted with a yellow box) containing the residues important for SARS PLpro interaction to K48-Ub_2_ and ISG15 binding is highly divergent between PLpro's from SARS and HKUs. The structure elements were generated using the X-ray crystal structure of SARS PLpro (pdb: 2FE8).
(TIF)

Table S1 Data collection and refinement statistics for PLpro-ubiquitin aldehyde complex.
(PDF)
