# Supplementary figures and images for: Structural Basis for the Ubiquitin-Linkage Specificity and deISGylating Activity of SARS-CoV Papain-Like Protease
Source: PLoS Pathog. 2014 May 22;10(5):e1004113. doi: 10.1371/journal.ppat.1004113 (PMC4031219; doi:10.1371/journal.ppat.1004113)

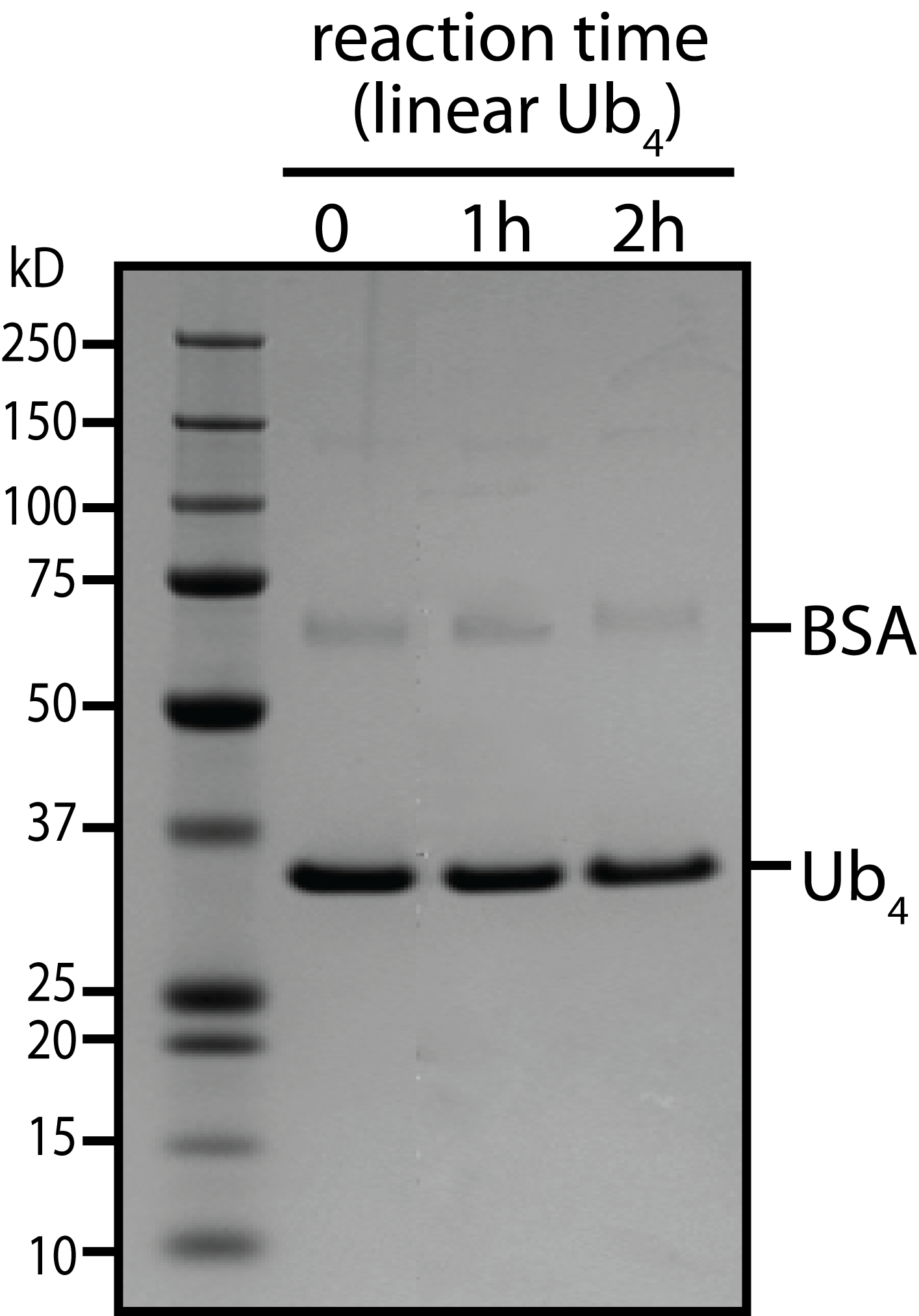

Supplement: Figure S1 — Processing of linear Ub4 by SARS-CoV PLpro. SARS-CoV PLpro (15 µM) was incubated with 3 µg of linear-Ub4 for 1 and 2 hours (h) at room temperature. The reaction was quenched with SDS sample buffer containing 250 mM Tris, pH 6.8, 10% SDS, 50% glycerol, 0.02% bromophenol blue and 35 mM beta-mercaptoethanol. Protein bands corresponding to Ub and BSA are shown. The molecular weight marker is shown in kD. No cleavage of linear Ub4 by SARS-CoV PLpro is detected. (TIF) [file ppat.1004113.s001.tif]

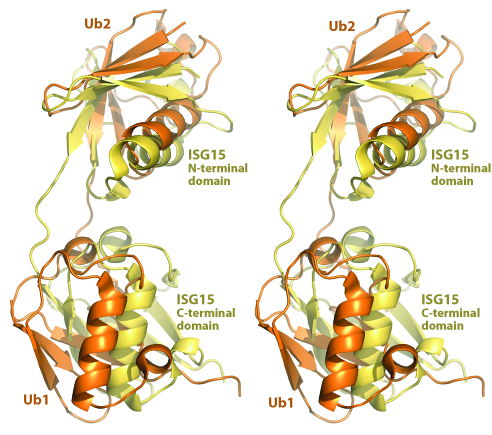

Supplement: Figure S2 — ISG15 resembles a di-ubiquitin molecule. Overlay of K48-Ub2 (orange) and ISG15 (yellow) rendered a Cα RMSD of 6.32 Å (147 to 147 atoms). The two distal regions involved in binding Ub2 or ISG15 are labeled as Ub distal-1 (closest to active site, location of single ubiquitin binding) or ISG15 C-terminal domain and Ub distal-2 (binding of second ubiquitin-like domain) or ISG15 N-terminal domain. (TIF) [file ppat.1004113.s002.tif]

**a**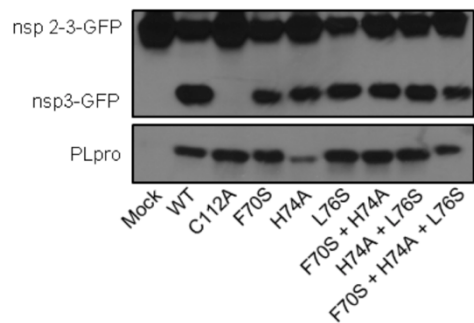**b**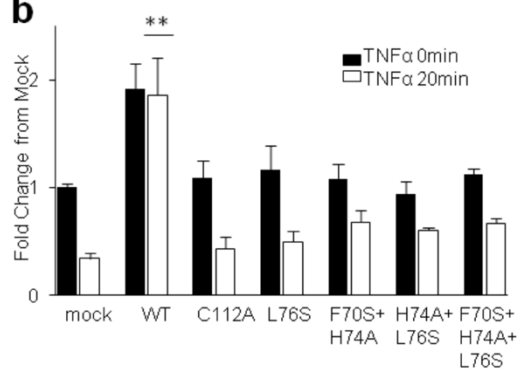**c**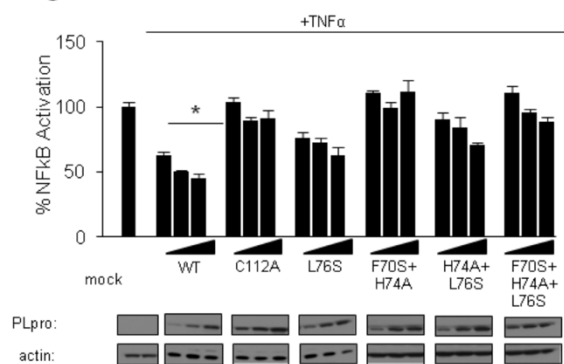

Supplement: Figure S3 — (A) HEK293 cells were transfected with constructs expressing nsp2-3-GFP and SARS-CoV PLpro-V5 wild type, catalytic mutant (C112A) or ridge mutants. Cells were incubated for 24 hours at 37°C and then lysed with lysis buffer A. Lysates were run on 10% SDS-PAGE and Western blot was performed using anti-GFP and anti-V5. (B) Quantification of IκBα using Fluorchem E System and AlphaView software (Protein Simple). ** = The levels of IkB-HA were significantly increased in the presence of WT PLpro compared to mock, C112A, and F70S (p<0.05) by mixed ANOVA and there was no decrease after TNFα treatment (p = 0.675) by Dunnet t-test. (C) 293HEK cells were transfected with a construct containing a firefly luciferase reporter driven by an NFkB dependent promoter and a Renilla luciferase under control of a constitutive promoter. After 12 hours, TNFα was added to a final concentration of 10 ng/mL and the cells were incubated for an additional 4 hours. Cells were lysed in passive lysis buffer and 25 ul of lysate was used in Promega's Dual Luciferase Reporter Assay. Results are normalized to induction of NFkB reporter activity by TNFα. Panels below are western blots of the lysates using anti-V5 for detection of PLpro and anti-actin as a protein loading control. Experiments were performed in triplicate and repeated twice. * = p<0.05 statistical difference from mock transfected cells by student t-test. (PDF) [file ppat.1004113.s003.pdf]

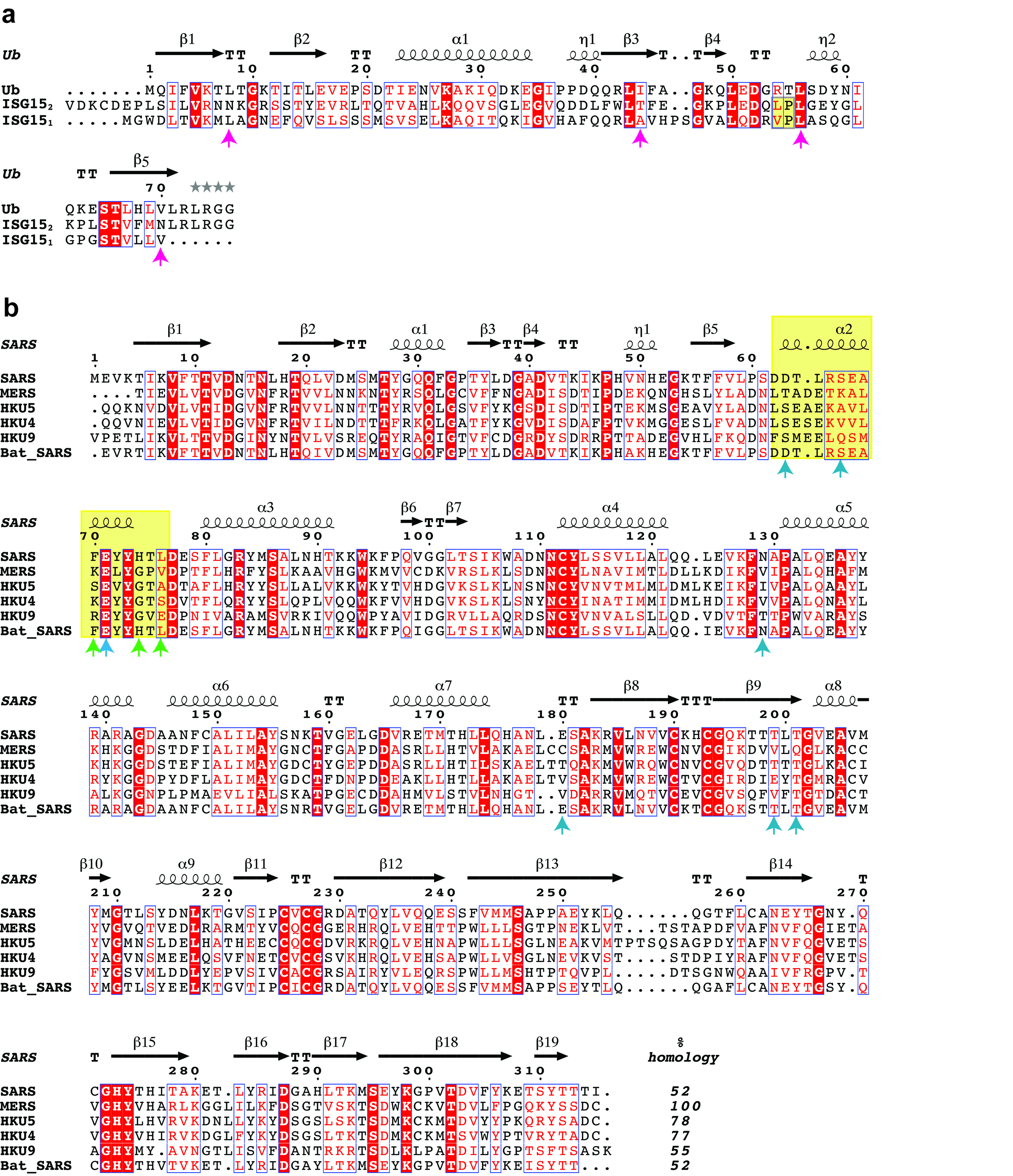

Supplement: Figure S4 — Multiple sequence alignments presenting the secondary structure elements on top: α-helices (squiggles), β-strands (black arrows) and turn (TT). Highlighted are the highly conserved areas (blue boxes) containing the conserved residues (red boxes), homologous residues (red font), and divergent residues (black font). (A) Comparison of the amino acid sequence between the β-grasp domain of ubiquitin to each β-grasp domain of ISG15. The residues comprising the ubiquitin and ISG15 hydrophobic patch are highlighted with a magenta arrow and a yellow box, respectively. The structure elements were generated using the X-ray crystal structure of ubiquitin (pdb: 1UBQ). (B) The papain-like protease (PLpro) domain from the beta coronavirus 2b (SARS and bat-SARS), 2c (bat-HKU4 and 5) and 2d (HUK9) share high amino acid sequence homology. SARS PLpro residues identified by site-directed mutagenesis as important for K48-Ub2 and ISG15 binding are highlighted with green arrows, while those that did not seemed to be important are highlighted with blue arrows. The α-helix 2 (highlighted with a yellow box) containing the residues important for SARS PLpro interaction to K48-Ub2 and ISG15 binding is highly divergent between PLpro's from SARS and HKUs. The structure elements were generated using the X-ray crystal structure of SARS PLpro (pdb: 2FE8). (TIF) [file ppat.1004113.s004.tif]
