# Supplementary material for: Structural Basis for the Ubiquitin-Linkage Specificity and deISGylating Activity of SARS-CoV Papain-Like Protease
Source: PLoS Pathog. 2014 May 22;10(5):e1004113. doi: 10.1371/journal.ppat.1004113 (PMC4031219; doi:10.1371/journal.ppat.1004113)
Supplement: Table S1 — Data collection and refinement statistics for PLpro-ubiquitin aldehyde complex. (PDF) [file ppat.1004113.s005.pdf]

Table 1. Data Collection and Refinement Statistics for PLpro-Ubiquitin aldehyde complex

**Data Collection Parameters**	
Crystal Conditions	Flash-Cooled at 100 ^{o}K
X-ray Source and Detector	LS-CAT Beamline 21-ID-F, MarMosaic 225
X-ray Wavelength (Å)	0.97872
Resolution Limit (Å)	2.75
Space Group	P 31 2 1
Unit Cell Dimensions	
a, *b*, *c* (Å)	45.15, 45.15, 342.66
α, β, γ (°)	90, 90, 120
**Data Processing Statistics**	**Overall [Last Shell]**
Data Resolution Range (Å)	100 - 2.75 Å [2.80-2.75]
Mosaicity	
Reflections	
Total Recorded (n)	92,187
Unique Recorded (n)	12,019
Completeness (%)[a]	99.0 [100.0]
Average Redundancy	
R_{merge} (%)	6.3 [14.1]
Average $I/\sigma I$	37.3 [16.4]
Wilson *B*-factor (Å^2)	42.2
**Refinement Statistics**	**Overall [Last Shell]**
Data Resolution Range (Å)	39.7 - 2.75 Å [3.03-2.75]
Reflections in Working Set (n)	11,395
Reflections in Test Set (n)	575 (5.0%)
R_{work} (%)[b]	18.7 [23.0]
R_{free} (%)[c]	27.9 [35.1]
Figure of merit[d]	0.88 [0.73]
RMS Deviations	
Bond length (Å)	0.012
Bond angles (^{o})	1.09
Ramachandran Plot	
Most Favored (%)	92
Allowed	7
Outliers (%)	1
**Average *B*-factor (Å^2)**	
Protein (Chains A + B)	44.0
Ubiquitin (Chain A)	
PLpro (Chain B)	
Solvent H_2O (101)	27.4

[a] Completeness for $I/\sigma(I) > 1.0$.
[b] $R_{work} = \sum ||F_o|-|F_c|| / \sum | F_o |$
[c] R_{free} was calculated against 5% of the reflections removed at random.
[d] Figure of merit = $(| \sum P(\alpha)e^{i\alpha} / \sum P(\alpha) |)$, where α is the phase and $P(\alpha)$ is the phase probability distribution.
